# Novel Technique for Hand-Assisted Laparoscopic Nephrectomy for Advanced Renal Cell Carcinoma with Renal Vein and Inferior Vena Cava Thrombi: Three Case Reports

**DOI:** 10.1155/2022/8177947

**Published:** 2022-12-28

**Authors:** Yuichi Machida, Hiroki Yoshiuchi, Yuko Kitano, Yoshikazu Kuroki, Masato Kamizuru, Junji Uchida

**Affiliations:** ^1^Department of Urology, Osaka Metropolitan University Graduate School of Medicine, 1-4-3 Asahi-machi, Abeno-ku, Osaka 545-8585, Japan; ^2^Department of Urology, Yao Municipal Hospital, 1-3-1, Ryuge-cho, Yao-City, Osaka 581-0069, Japan

## Abstract

**Introduction:**

The treatment of thrombi in the renal vein (RV) and inferior vena cava (IVC) requires advanced laparoscopic experience. We present three cases of hand-assisted laparoscopic nephrectomy (HALN) using a novel technique for treating advanced renal cell carcinoma (RCC) with thrombi in the RV and IVC. *Case Presentation*. Three patients with RCC with RV or IVC thrombus below level I underwent HALN. Two patients had right RCC with RV and IVC thrombi. One patient had left RCC with an RV thrombus. We hooked a vessel loop to the end of the thrombus and pulled it up manually to make space for vascular processing. The RV was narrowed and dissected using Hem-o-lok clips or an Endo GIA stapler.

**Conclusion:**

In carefully selected cases, renal vascular processing could be easily and safely performed using a vessel loop in HALN with thrombectomy.

## 1. Introduction

In 2000, Savage and Gill reported the first laparoscopic radical nephrectomy (LRN) for a case of renal cell carcinoma (RCC) with thrombus extending into the renal vein (RV) [1]. To date, multiple case reports and case series have been presented on the successful use of pure laparoscopic, laparoscopic-assisted, or hand-assisted LRN and thrombectomy. These studies used the Satinsky vascular clamp or laparoscopic bulldog clamp to control the inferior vena cava (IVC), which requires experience in laparoscopic vascular suturing.

Reports have shown that hand-assisted laparoscopic nephrectomy (HALN) has the advantages of shorter operative time and fewer complications than LRN [2–4]. However, if the RV does not have enough space for vascular clips or endoscopic stapling devices, laparoscopic IVC suturing must be performed by placing a laparoscopic clamp or an open conversion, which reduces the advantages of HALN.

Here, we report the results of a case series of the modified HALN technique with vascular loops in patients with advanced RCC with RV and IVC thrombi below level I.

## 2. Case Presentation

Three patients with RCC with RV or IVC thrombus below level I underwent HALN at Yao Municipal Hospital between January 2016 and December 2020. Tumor thrombus level was defined according to the Mayo classification of macroscopic venous invasion in RCC, with level I defined as a thrombus either at the entry of the RV or within the IVC less than 2 cm from the confluence of the RV and IVC. The same surgeon performed all surgeries.

**Figure 1:**
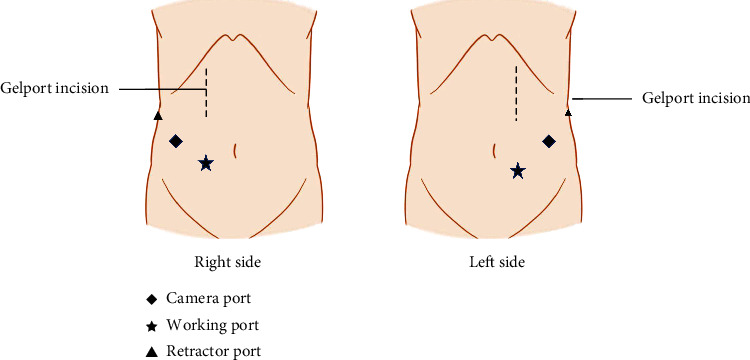
Placement of the hand port and trocars.

The patient was placed in the lateral decubitus position. The port layout is shown in Figure 1. An 8 cm incision was made from below the costal arch along the outer edge of the rectus abdominis muscle through which the GelPort® was inserted. The incision position of the hand port was higher than that in conventional HALN [5]. This modification allowed the intra-abdominal hand to be closer to the renal hilum for easier handling.

**Figure 2:**
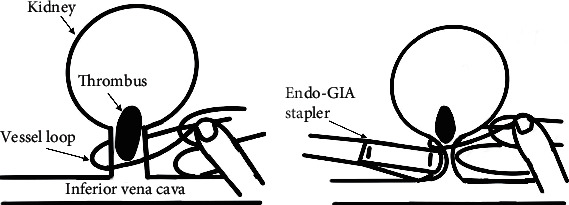
Schema of vascular processing in hand-assisted laparoscopic nephrectomy. (a) Thrombus tip is hooked using a vessel loop. (b) Thrombus is pulled up using the vessel loop, creating space for it to be processed using the Endo GIA stapler.

The schema of vessel processing using a vessel loop is shown in Figure 2. A laparoscopic retractor was used to elevate the kidney from the caudal side, and the renal artery was ligated using Hem-o-lok® clips before approaching the RV. Doppler ultrasound was performed to identify the end of the tumor thrombus. We hooked a vessel loop to the end of the thrombus and pulled it up manually to make space for vascular processing. The RV was narrowed and dissected using an Endo GIA stapler or Hem-o-lok clips. The specimen was removed from the hand port incision.

**Figure 3:**
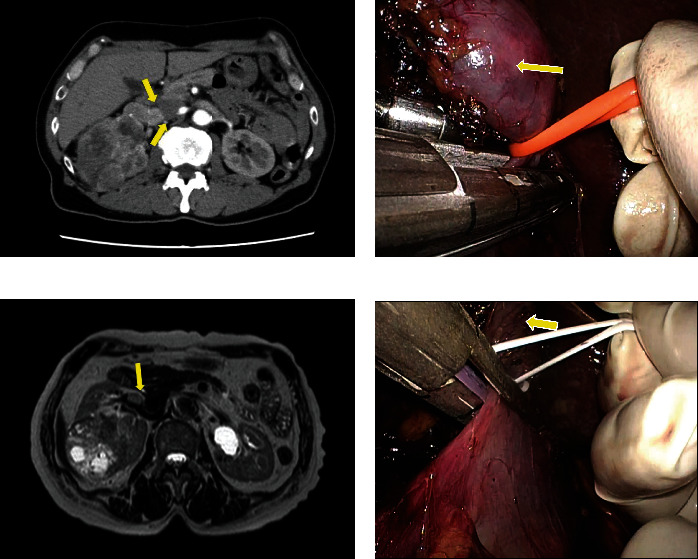
Imaging and intraoperative findings of the cases of right renal cell carcinoma (RCC) with thrombus ((a, b) case 1 and (c, d) case 2). (a) Computed tomography demonstrated a renal tumor thrombus extending 0.5 cm into the inferior vena cava. (b) The tumor was pulled up within the renal vein using the vessel loop, and the renal vein was divided using the Endo GIA stapler. (c) Magnetic resonance imaging demonstrated a renal tumor thrombus within the renal vein. (d) The tumor was pulled up using the vessel loop, and the renal vein was divided using the Endo GIA stapler. Arrowheads indicate the tip of the tumor thrombus.

**Figure 4:**
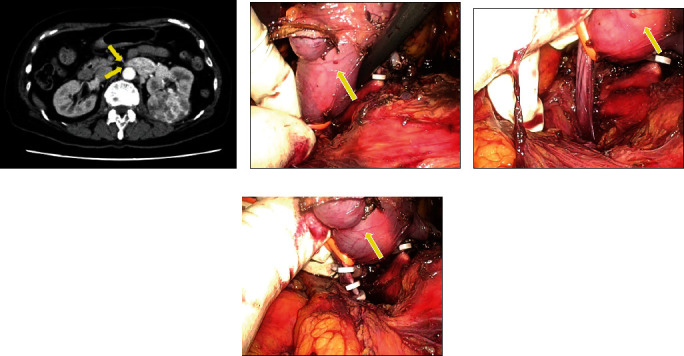
Imaging and intraoperative findings of the cases with left renal cell carcinoma with thrombus (case 3). (a) Computed tomography demonstrated a thrombus extending 2 mm beyond the right aortic border. (b) A vessel loop hooked to the thrombus tip. (c) The thrombus was pulled up using a vessel loop. (d) The left renal vein was dissected using Hem-o-lok clips. Arrowheads indicate the tip of the tumor thrombus.

Figure 3 shows computed tomography (CT) or magnetic resonance imaging and intraoperative findings of the cases with right RCC (cases 1 and 2), and Figure 4 shows those of the case with left RCC (case 3). In case 1, CT demonstrated a tumor thrombus extending 0.5 cm into the IVC. In case 3, the thrombus extended 2 mm beyond the right aortic border to within 1.5 cm of the left border of the IVC. In all cases, the thrombi could be pulled up proximally using a vessel loop, facilitating subsequent vascular ligation with an Endo GIA stapler or Hem-o-lok clips. The procedure was successful in all patients without open conversion or complications. All patients had negative resection margins.

## 3. Discussion

In this study, we demonstrated our modified HALN technique using a vessel loop in patients with RCC with RV and IVC thrombi below level I. The greatest advantage of this novel technique may be that the end of the thrombus can be hooked onto the vessel loop and pulled up while elevating the renal hilus via delicate manipulation of the fingertips. An intra-abdominal hand improves tactile feedback to the surgeon, thereby lowering the threshold for laparoscopic surgery [5]. Since adhesions around the renal hilus are expected in such an invasive cancer, inadvertent manipulation using forceps may lead to bleeding, so that manipulation by blunt dissection of the fingertips might be the optimal technique to avoid surgical complications. This novel technique may allow simpler and less invasive vascular treatment of the RV with thrombus. Furthermore, if the tumor thrombus is inadequately resected by our method, or if there is bleeding due to inadequate vascular control, the position of our hand port allows easy access to the renal vessels, making it easy to treat the vessels under direct vision through the hand port incision even when laparoscopic techniques are difficult to perform.

Although the dissection of the RV with thrombus has been reported to be possible in pure laparoscopic surgery by forceps manipulation, milking the thrombus and clipping the RV are difficult, and securing the field of view in the renal hilum is more difficult than that in HALN. Moreover, HALN has been reported to have advantages when the tumor is massive or the patient's medical complications require a prompt response; HALN may avoid unnecessary conversion to open surgery [6].

However, there might be significant limitations in the use of this novel HALN technique. It can only be used for treating RCC with thrombus that has little progression to the IVC. If a vessel loop cannot be hooked to the edge of the thrombus because of thrombus development, vascular dissection using this procedure is impossible. In such cases, the IVC can be clamped laparoscopically using a bulldog or Satinsky clamp and closed by running a 3-0 polypropylene suture after tumor removal, requiring a more complex technique [7, 8]. Kovac and Luke reported that for a case of right RCC with a 1 cm thrombus extending into the IVC undergoing HALN, lateral traction by hand was applied to the right kidney, allowing the thrombus to be pulled into the RV and away from the IVC [9]. In this study, a laparoscopic retractor was used to elevate the kidney from the caudal side, allowing the thrombus to return into the RV. However, in cases where the thrombus does not return to the RV after such a procedure, the aforementioned suture technique is required, and the surgeon must be proficient in laparoscopic suture techniques.

## 4. Conclusions

We demonstrated that HALN is technically safe and effective for renal tumors with thrombus below level I. The use of a vessel loop in dissecting the RV with thrombus is easier to divide with an endovascular stapler or Hem-o-lok clips than previously reported techniques.
